# Hydrogen Gas Mitigates Acute Hypoxia-Induced Oxidative and Inflammatory Brain Injuries in Medaka (*Oryzias latipes*)

**DOI:** 10.3390/antiox14091130

**Published:** 2025-09-18

**Authors:** Eriko Sato, Naohiro Shimamura, Chikako Saiki, Katsuhisa Sunada, Nobuhiko Miwa, Li Xiao

**Affiliations:** 1 Department of Physiology, School of Life Dentistry at Tokyo, The Nippon Dental University, 1-9-20 Fujimi, Chiyoda-ku, Tokyo 102-8159, Japanchikako@tky.ndu.ac.jp (C.S.); 2 Department of Dental Anesthesiology, School of Life Dentistry at Tokyo, The Nippon Dental University, Tokyo 102-8159, Japan; shimamura@tky.ndu.ac.jp (N.S.); ksunada@tky.ndu.ac.jp (K.S.); 3 Incorporated Association Hydrogen Medical Institute, Minatojima Minamicho 1-6-4, ChuOh-Ku, Kobe 650-0047, Japan; vitamin2002rejuvenation@yahoo.co.jp; 4 Faculty of Life Sciences, Prefectural University of Hiroshima, Hiroshima 727-0023, Japan

**Keywords:** hydrogen therapy, oxidative stress, inflammation, hypoxia, medaka fish, 8-OHdG, neuroprotection, antioxidant capacity

## Abstract

Hypoxia-induced oxidative stress and inflammation in the brain are critical contributors to neurological disorders. Hydrogen gas has emerged as a therapeutic agent with potent antioxidant and anti-inflammatory properties. In this study, we evaluated the protective effects of hydrogen against acute hypoxia-induced brain injuries in medaka. Fish were exposed to hypoxia and then recovered in water bubbled with air, hydrogen, or ozone. LOX-1 hypoxia probe imaging and HIF-1α immunostaining showed persistent tissue hypoxia in the air and ozone groups, which was significantly reduced by hydrogen treatment. Histological analysis revealed extensive vascular congestion in the midbrain after hypoxia, which was markedly alleviated by hydrogen. TUNEL assay demonstrated that hydrogen suppressed hypoxia-induced neuronal apoptosis. Immunohistochemistry and ELISA showed elevated levels of 8-hydroxy-2′-deoxyguanosine (8-OHdG) and proinflammatory markers (COX-2, IL-6, TNF-α) in the brains of air- and ozone-treated fish; these increases were significantly attenuated by hydrogen. ORAC assay confirmed that hydrogen restored brain antioxidant capacity. Behavioral analysis further demonstrated that hydrogen treatment improved locomotor activity and stabilized respiratory function. These results indicate that hydrogen protects medaka against hypoxia-induced oxidative and inflammatory injuries and may represent a promising therapeutic strategy for hypoxia-related neurological disorders.

## 1. Introduction

Oxygen is indispensable for maintaining neuronal metabolism and synaptic function. Even brief episodes of hypoxia can cause widespread dysfunction in the brain, one of the most oxygen-dependent tissues in the body [1]. Cerebral hypoxia disrupts mitochondrial respiration, elevates the production of reactive oxygen species (ROS), depletes endogenous antioxidant defenses, and destabilizes cellular energy homeostasis. These effects collectively trigger oxidative stress and neuroinflammation, leading to neuronal dysfunction and death [2,3,4].

Hypoxia-induced oxidative brain damage is a key contributor to both acute neurological conditions, such as stroke and perinatal asphyxia [5,6], and chronic neurodegenerative diseases associated with aging. Sustained or intermittent hypoxia promotes oxidative DNA damage, lipid peroxidation, and protein oxidation, which in turn trigger neuronal apoptosis, impair neurogenesis, and contribute to cognitive decline [7,8,9]. Elevated levels of 8-hydroxy-2′-deoxyguanosine (8-OHdG), a marker of oxidative DNA damage, have been detected in brain regions under chronic intermittent hypoxia, related to early-stage neurodegeneration, underscoring the role of oxidative stress in hypoxia-related neuropathology [10].

A central player in the cellular response to hypoxia is hypoxia-inducible factor-1 alpha (HIF-1α), a key transcriptional regulator that enables cellular adaptation to low oxygen. In neonatal hypoxic–ischemic brain injury, transient HIF-1α activation promotes neuroprotection through the induction of erythropoietin (EPO) and vascular endothelial growth factor (VEGF), supporting neuronal survival and promoting neovascularization. However, prolonged HIF-1α expression has been associated with increased neuronal apoptosis and disruption of the blood–brain barrier (BBB), thereby exacerbating neuroinflammation and tissue damage [11,12].

In addition, hypoxia stimulates the expression of pro-inflammatory enzymes and cytokines, including cyclooxygenase-2 (COX-2), interleukin-6 (IL-6), and tumor necrosis factor-alpha (TNF-α), which amplify neuroinflammatory cascades and accelerate neuronal injury [13,14,15]. Vascular dysfunction is also a hallmark of hypoxic brain injury, with hypoxia compromising BBB integrity, inducing microvascular hemorrhage, and promoting cerebral edema [16]. These vascular pathologies impair clearance of neurotoxic metabolites and further propagate neurodegeneration.

Given the multifactorial nature of hypoxia-induced brain injury—including excessive production of reactive oxygen species (ROS), inflammatory signaling, and vascular dysfunction—there is a pressing need for therapeutic strategies that can target these interconnected pathological pathways. Molecular hydrogen (H_2_) has gained increasing attention as a potential neuroprotective agent due to its selective antioxidant and anti-inflammatory properties. Preclinical studies in animal models of stroke, neonatal hypoxic–ischemic encephalopathy, and traumatic brain injury have demonstrated that H_2_ administration reduces infarct size, suppresses neuronal apoptosis, attenuates cytokine release, and improves functional outcomes [17]. For instance, inhalation of 2% H_2_ for one hour significantly mitigated brain injury and cognitive impairment in mice by modulating neuroinflammation, reducing oxidative stress, and preventing neuronal cell death; similar protective effects were observed in the lungs following traumatic brain injury [18,19]. Moreover, a recent prospective, multicenter, randomized, double-blind, placebo-controlled clinical trial showed that inhalation of 2% H_2_ improved neurological outcomes in patients following brain ischemia during post-cardiac arrest care [20].

Medaka is a genetically tractable and physiologically relevant vertebrate model that shares conserved hypoxia signaling pathways with mammals, making it well-suited for studying the effects of oxygen deprivation. Previous studies have demonstrated that hypoxic stress in medaka elicits broad biological responses, including altered gene expression, disruption of follicular development, hormonal imbalances, and even shifts in sex differentiation from genetic females to phenotypic males [21,22]. At the molecular level, hypoxia activates key neurovascular and metabolic pathways such as p53 and PI3K–Akt, further emphasizing the suitability of medaka for hypoxia-related research [23]. For neurological studies, medaka offers additional advantages due to its transparent embryos, conserved nervous system, measurable behaviors, and ethical and practical benefits. Our study further highlights the importance of optimized tissue preservation techniques in maximizing the utility of medaka in neurobiological investigations [24].

Building on these insights, the present study utilizes adult Japanese medaka (*Oryzias latipes*) to model acute hypoxia-induced injury in the brain. We examined multiple pathological outcomes, including oxidative stress, inflammation, vascular congestion, neuronal apoptosis, altered respiratory patterns, and behavioral deficits. Leveraging the medaka’s small size and suitability for whole-organism analysis, we established a simple and reproducible protocol for inducing acute hypoxia and evaluating recovery. A comprehensive set of assessments—including histological staining, TUNEL(terminal deoxynucleotidyl transferase dUTP nick end labeling) assay, immunohistochemistry (IHC), oxidative DNA damage detection, enzyme activity measurements, respiratory rate monitoring, and behavioral tracking—were employed to characterize tissue injury and the effects of recovery interventions. Additionally, we investigated the therapeutic potential of hydrogen gas (H_2_), a small molecule with known antioxidant and anti-inflammatory properties, by comparing post-hypoxia recovery outcomes in fish exposed to hydrogen-, air-, or ozone-bubbled water. This study presents a novel application of the medaka model for studying hypoxia-induced organ damage and highlights hydrogen gas as a promising intervention for brain injury related to acute hypoxic stress.

## 2. Materials and Methods

### 2.1. Reagents and Instruments

The Hypoxia Probe Solution (LOX-1) was purchased from MEDICAL & BIOLOGICAL LABORATORIES CO., LTD. (Tokyo, Japan). Davidson’s solution was obtained from Muto Pure Chemicals Co. Ltd. Other general reagents were sourced from FUJIFILM Wako Pure Chemical Corporation (Osaka, Japan). A dissolved oxygen meter (DO-5519E) was obtained from Kenis co., Inc. (Osaka, Japan). A hydrogen gas generator (Lita Air) was obtained from Lita Heart International Co., Ltd. (Osaka, Japan), and the ozone gas generator (R200-Black-JP, WUOAUM) and air pump (FEDOUR 300LPH) were purchased from Amazon.co.jp (Amazon Co., Tokyo, Japan).

### 2.2. Animals

All procedures were approved by the Animal Ethics Committee of the Nippon Dental University School of Life Dentistry at Tokyo (authorization number: 24-14-1, 10 October 2024) and were conducted in accordance with the ARRIVE guidelines. Wild-type adult Japanese medaka (*Oryzias latipes*), including Matsushiro, Himedaka (red), and black medaka strains, was obtained from the NBRP Medaka Bioresource Project (https://shigen.nig.ac.jp/medaka/, accessed 14 January 2025) and a local aquarium supplier. Fish were housed in aquaria under a 12-h light/12-h dark cycle in dechlorinated tap water (sodium thiosulfate, 2 mL/10 L) at room temperature and fed daily with commercial dry feed. Experimental fish (mixed sex, 30–40 mm total length) were randomly assigned to treatment groups. As no significant sex-specific differences were observed, data from males and females were pooled for analysis.

### 2.3. Hypoxia Exposure and Recovery

**Figure 1:**
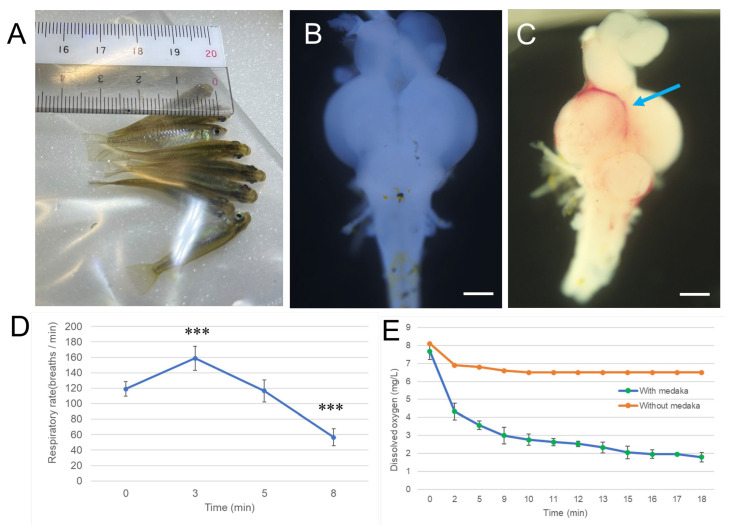
Hypoxia induces changes in respiratory rate and brain hemorrhage in medaka. Matsushiro medaka was exposed to acute hypoxia by placement in a Ziplock bag containing minimal water and air for 9 min. Opercular movements (breathing rate) were recorded during the exposure using a TOMLOV digital microscope (Supplemental Video S1). The number of breaths for each fish was counted over 30-s intervals, repeated three times at various time points. Dissolved oxygen was measured by inserting a probe into the same bag, which was further sealed within two larger Ziplock bags to prevent air leakage. Oxygen levels were continuously monitored for 18 min, and a representative 10 s segment from 8–9 min is shown in Supplemental Video S1. Following hypoxia, fish were transferred to air-bubbled water and allowed to recover for approximately one hour. Whole brains were then excised and examined macroscopically. (**A**), Photograph of medaka undergoing hypoxia exposure in a Ziplock bag. (**B**), Representative image of a whole brain without hypoxia exposure. (**C**), Representative image of a whole brain after recovery from hypoxia. Blue arrow indicates areas of hemorrhage. Scale bar = 500 µm. (**D**), Changes in respiratory rate during hypoxia exposure. *** *p* < 0.001 vs. 0 min. (**E**), Dissolved oxygen levels during hypoxia exposure. Experiments were independently repeated five times.

To induce hypoxia, 5–20 medaka was placed in sealed Ziplock bags (A7–A5 size) containing a small volume of water and limited air for 9–30 min (Figure 1A). Opercular movements were recorded using a TOMLOV digital stereo microscope (TOMLOV co. Shenzhen, China). Dissolved oxygen was measured by using the dissolved oxygen meter (Supplemental Video S1). Following hypoxia, fish were recovered for 1 h in water bubbled with air (Air), 4% hydrogen in air (H_2_), or ozone gas (O_3_, 0.02–0.03 ppm). Fish were euthanized by cryogenic anesthesia, as previously described [25], and brains were harvested for further analysis or fixed in Davidson’s solution, dehydrated, and paraffin-embedded.

### 2.4. LOX-1 Signal Imaging

LOX-1 (2 mM, 10 µL) was administered via intraperitoneal injection under cryogenic anesthesia using a microsyringe pump (MSPE-1, As One, Tokyo, Japan) [25]. After 1 h of recovery in normal water, fish underwent hypoxia and recovery as described. Brains were sectioned (200 μm) using a vibratome (NeoLinearSlicer MT, Dosaka EM Co., Kyoto, Japan) and imaged with a laser scanning microscope (LSM700, Carl Zeiss, Tokyo, Japan). Fluorescence intensity was quantified with an ImageJ software (version 1.54p, National Institutes of Health, Bethesda, MD, USA) [26].

### 2.5. Histology and Immunohistochemistry

Paraffin-embedded brain sections (10 μm) were subjected to hematoxylin and eosin (H&E) and immunohistochemical staining. Primary antibodies included anti-HIF-1α (H1alpha67, Thermo Fisher Scientific, Tokyo, Japan), anti-8-OHdG (Nikken SEIL Co., Ltd., Shizuoka, Japan), and anti-COX-2 (ab62331, Abcam, Tokyo, Japan). HRP-conjugated secondary antibodies were from Abcam (ab6802, ab16284). The DAB reaction was performed using a HISTOFINE SAB-PO (M) kit (Nichirei Bioscience Inc., Tokyo, Japan). Imaging was conducted with a NanoZoomer S20 (Hamamatsu Photonics K. K., Shizuoka, Japan) and quantified using ImageJ.

### 2.6. TUNEL Assay

Apoptotic cells were detected using the In Situ Apoptosis Detection Kit (Takara bio Inc., Shiga, Japan). Sections were deparaffinized, treated with proteinase K (Sigma-Aldrich, Tokyo, Japan), and permeabilized. Samples were labeled with TdT enzyme at 37 °C for 1 h, counterstained with propidium iodide (PI), and imaged with a laser scanning microscope (LSM700, Carl Zeiss, Tokyo, Japan). Positive cells were quantified using ImageJ.

### 2.7. ELISA

Proteins from medaka brain tissues were extracted using EzRIPA buffer (ATTO, Co., Tokyo, Japan). Following preparation, protein samples from medaka brain tissues were analyzed using ELISA kits for mouse IL-6 and TNF-alpha (M6000B, MTA00B, Bio-Techne, Tokyo, Japan) according to the manufacturers’ instructions.

### 2.8. Acetylcholinesterase (AChE) Activity Assay

Brain tissues from medaka were excised on ice, washed with PBS (−), and homogenized in 300 μL of ice-cold 0.05 M phosphate buffer. The homogenates were centrifuged at 10,000× *g* for 10 min at 4 °C, and the resulting supernatants were collected. Protein concentrations were measured using a BCA assay. Supernatants with normalized protein levels were used for catalase and AChE activity assays, as well as for the ORAC assay.

AChE activity was measured using a fluorometric assay [27]. Supernatants were incubated with 5.6 mM acetylthiocholine iodide in Buffer I (0.12 M NaCl, 0.2% Triton X-100, 1 mM EDTA, and 50 mM HEPES, pH 7.5) for 30 min at room temperature. A 20 μL aliquot of the reaction mixture was transferred to a 96-well plate and mixed with Buffer II (1 mM EDTA, 0.2% Triton X-100, and 50 mM acetate buffer, pH 5.0) and 20 μL of 7-diethylamino-3-(4-maleimidophenyl)-4-methylcoumarin (0.4 mM in acetonitrile). After incubation for 1 h at room temperature, fluorescence intensity was measured at an excitation/emission wavelength of 365/450 nm using a microplate reader (HITACHI SH-9000Lab).

### 2.9. Oxygen Radical Absorbance Capacity (ORAC) Assay

To evaluate the antioxidant capacity of brain tissues, the ORAC assay was performed as previously described [28]. Briefly, 25 μL of brain tissue supernatant or Trolox standard was mixed with 25 μL of 12 mM AAPH and 150 μL of 70 nM fluorescein in each well of a black 96-well microplate. Fluorescence was measured every 5 min for 90 min at 37 °C (excitation: 480 nm; emission: 520 nm) using a microplate reader (HITACHI SH-9000Lab).

### 2.10. Movement Tracking of Medaka

To assess hypoxia-induced behavioral changes, recovery-phase videos from each group were analyzed using Tracker software (version 6.3.2) (https://opensourcephysics.github.io/tracker-website/, accessed 10 July 2025). After spatial and temporal calibration, individual fish were tracked to measure swimming distance, as well as the frequency of opercular (gill) movements. These parameters were used to evaluate locomotor activity and respiratory response during the recovery phase.

### 2.11. Statistical Analysis

Statistical analysis was conducted following the methods outlined in our previous report [24,29]. All data were analyzed using GNU PSPP Statistical Analysis Software (version 0.8.2-gad9374) (https://www.gnu.org/software/pspp/, accessed 11 March 2025) and EZAnalyze Excel-based tools (http://www.ezanalyze.com/, accessed 11 March 2025). One-way analysis of variance (ANOVA) was performed, followed by post hoc tests, including Tukey’s test and Bonferroni correction. Statistical significance was defined as *p* < 0.05. All experiments were independently repeated 3 to 5 times.

## 3. Results

### 3.1. Exposure Duration of Hypoxia and Severity of Brain Damage in Medaka

We first investigated the relationship between hypoxia exposure time and the severity of brain damage in medaka. Because changes in respiratory rate are a simple and observable indicator of hypoxia severity in medaka, we first analyzed hypoxia-induced respiratory changes in parallel with dissolved oxygen levels. As shown in Figure 1 and supplemental Video S1, medaka was placed in a Ziplock bag with minimal water and air to induce hypoxia. Opercular movements and dissolved oxygen levels were then recorded during the hypoxic exposure. The baseline respiratory rate was 119 breaths per minute; this increased to 159 breaths per minute after 3 min of hypoxia exposure. At 5 min, the rate returned to near baseline levels. After 8 min, however, the respiratory rate significantly dropped to 57 breaths per minute (Figure 1D). Dissolved oxygen in the Ziplock bag without medaka decreased only slightly, from 8.1 mg/L at baseline to 6.8 mg/L at 5 min, stabilizing at 6.5 mg/L after 10 min. In contrast, in the presence of medaka, dissolved oxygen dropped sharply from 7.8 ± 0.45 mg/L at baseline to 3.6 ± 0.23 mg/L at 5 min, and further declined to 2.9 ± 0.35 mg/L at 9 min (Figure 1E). These findings indicate that hypoxia initially induces hyperventilation, followed by a sharp decline in respiratory activity as oxygen depletion becomes critical.

Furthermore, compared with the brains of non-exposed fish (Figure 1B), cerebral hemorrhage (Figure 1C) was observed in some fish after 9 min of hypoxia exposure, accompanied by occasional mortality. These findings suggest that 9 min of exposure in the Ziplock bag–induced hypoxic environment represents the minimum lethal threshold for medaka. Previous studies in zebrafish have shown that hypoxia-induced brain damage is time-dependent. For example, infarcts became visible in the optic tectum after 10 min of hypoxia and progressed to involve the entire brain after 35 min [30]. These infarcts can be visualized using TTC (2,3,5-Triphenyltetrazolium chloride) staining.

To apply a similar approach, we initially attempted TTC staining in medaka to assess brain infarction. However, as shown in Supplemental Figure S1, TTC staining was highly dependent on the structural integrity of the brain. In untreated fish, intact brains stained uniformly red, while mechanically damaged brains displayed pale or unstained areas. This suggests that TTC staining is not suitable for small and fragile medaka brains due to dissection-related artifacts.

We therefore used H&E staining and TUNEL assays to assess histological and apoptotic changes. As shown in Supplemental Figure S2A, prolonged hypoxia exposure (over 30 min) led to notable vascular dilation and congestion in the midbrain. TUNEL staining revealed numerous apoptotic cells in the optic tectum (Supplemental Figure S2B). These findings confirm that extended hypoxia causes significant brain injury, and that H&E and TUNEL staining are more reliable than TTC staining for assessing vascular and cellular damage in medaka.

Previous studies have reported that dissolved oxygen concentrations below 2.8 mg/L can cause mortality in fish [31]. In our experiments, oxygen level was 2.9 ± 0.35 mg/L at 9 min when fish were placed in a Ziplock bag to induce hypoxia. Considering the sharp decline in opercular rate after 8 min, as well as the occurrence of cerebral hemorrhage and occasional mortality after 9 min, we selected 9 min as the standardized hypoxia duration for subsequent experiments.

### 3.2. Hydrogen Gas Suppresses Hypoxia-Induced LOX-1 Signal and HIF-1α Expression and Alleviates Vascular Damage in the Medaka Brain

To evaluate whether H_2_ exerts a protective effect against hypoxia-induced brain damage in medaka, we examined its impact on hypoxia markers and vascular integrity.

**Figure 2:**
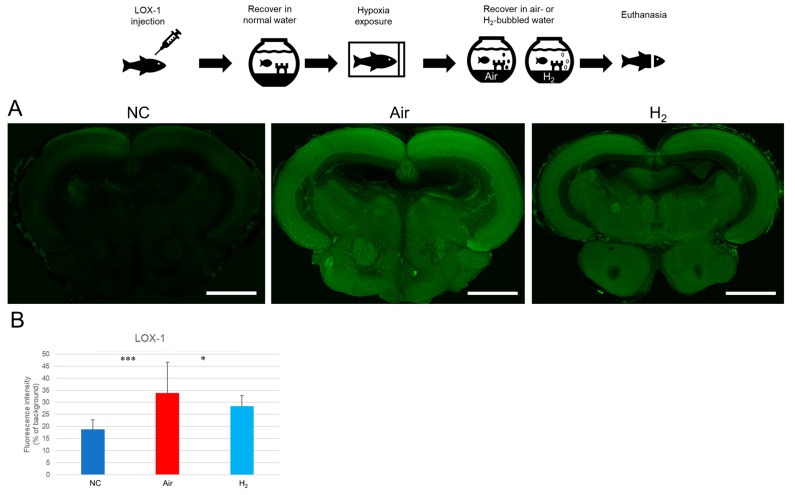
Effect of hydrogen gas on hypoxia-induced LOX-1 probe signal in medaka brain slices. Matsushiro medaka (*n* = 20/group) was injected with the LOX-1 hypoxia probe (2 mM, 10 µL) under cryogenic anesthesia and allowed to recover in normal breeding water for 1 h. The fish were then exposed to a hypoxic environment for 9 min, as described in the Materials and Methods. After hypoxia, they were immediately transferred to one of two recovery conditions for 1 h: air-bubbled water (Air) or hydrogen gas-bubbled water (H_2_). Negative control fish (NC) were kept in normal breeding water without hypoxia exposure. Following the treatment, the fish were euthanized, and their brains were sectioned at a thickness of 200 µm using a vibratome, then examined with a laser scanning microscope. The top panel illustrates the experimental procedure. (**A**), Representative images of LOX-1 fluorescence in medaka brain slices. Two additional examples for each treatment group are provided in Supplemental Figure S3. Scale bar = 500 µm. (**B**), Quantification of LOX-1 fluorescence intensity from 5 brain slices using ImageJ software. * *p* < 0.05, *** *p* < 0.001. Experiments were independently repeated three times.

We first assessed whether H_2_ can mitigate hypoxia itself by using a LOX-1 hypoxia probe. As shown in Figure 2A and Supplemental Figure S3, the midbrain of negative control (NC) fish exhibited only minimal LOX-1 signal, indicating normoxic conditions. In contrast, fish recovered in air-bubbled water (Air group) showed strong LOX-1 signals in the midbrain, suggesting substantial hypoxic stress. Notably, fish recovered in hydrogen gas-bubbled water (H_2_ group) displayed significantly reduced LOX-1 signal compared to the Air group (Figure 2B), indicating that H_2_ alleviated tissue hypoxia.

**Figure 3:**
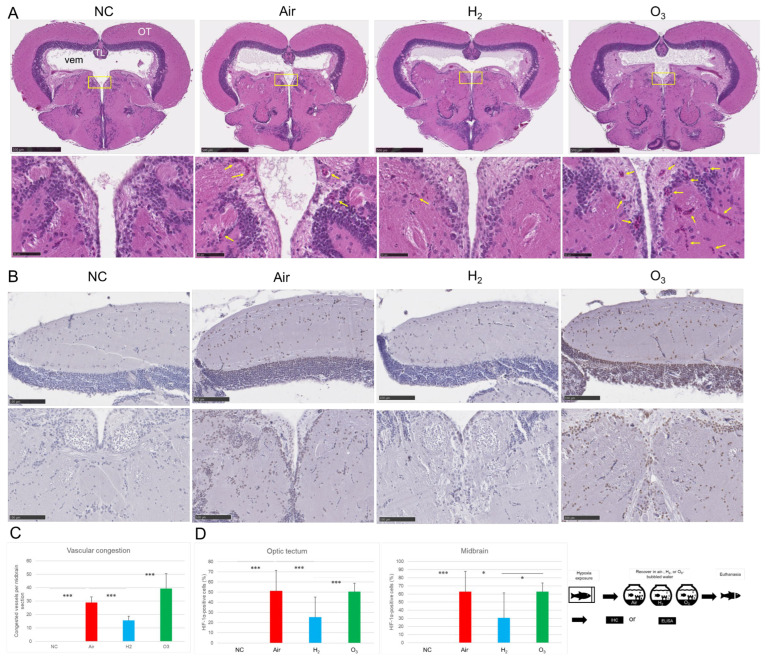
Effects of hydrogen gas on hypoxia-induced vascular congestion and HIF-1α expression in medaka brain tissues. Red medaka (*n* = 20 per group) was exposed to hypoxia as described in Materials and Methods. After exposure, fish were allowed to recover for 1 h in one of three conditions: Air, H_2_, or ozone gas-bubbled water (O_3_), as illustrated in the lower right panel (see Supplemental Video S2). Following recovery, fish were euthanized, and brains were fixed in Davidson’s solution, dehydrated, embedded in paraffin, and sectioned for histological analysis. Sections were stained with H&E or processed for IHC using an anti-HIF-1α antibody. Slides were scanned using a NanoZoomer S20 digital slide scanner. (**A**), Upper panels: representative H&E-stained brain sections. Two additional examples for each group are provided in Supplemental Figure S4. OT, optic tectum; TL, torus longitudinalis; Vem, ventriculus mesencephali. NC, the negative control. Scale bar = 500 µm. Lower panels: magnified views of boxed areas. Yellow arrows indicate congested blood vessels. Scale bar = 50 µm. (**B**), Representative IHC images showing HIF-1α expression. Two additional examples for each group are provided in Supplemental Figure S4. Brown indicates HIF-1α-positive cells; blue indicates nuclear counterstaining. Upper panels: optic tectum; lower panels: center of midbrain. Scale bar = 100 µm. (**C**), Quantification of the number of congested vessels per midbrain from 10 sections per group, analyzed using ImageJ. *** *p* < 0.001. (**D**), Quantification of HIF-1α-positive cells in the optic tectum and central midbrain region from 10 sections per group, analyzed using ImageJ. * *p* < 0.05, *** *p* < 0.001. Experiments were independently repeated five times.

Next, we examined the effect of H_2_ on HIF-1α expression and vascular congestion in the brain. Given that ozone gas (O_3_) is rich in oxygen, we included an O_3_-bubbled water group as a comparison. As shown in Figure 3A,C, H&E staining revealed widespread vascular congestion in the midbrain of the Air group compared to the negative control. In the H_2_ group, the number of congested blood vessels was significantly reduced. Surprisingly, the O_3_ group exhibited even more pronounced vascular congestion than the Air group.

Immunohistochemical analysis revealed no detectable HIF-1α expression in the NC group. In contrast, strong HIF-1α immunoreactivity was observed in both the optic tectum and central midbrain regions of the Air group, indicating substantial hypoxic stress. The O_3_ group exhibited a similar upregulation pattern, suggesting that ozone treatment did not alleviate hypoxia. Notably, the H_2_ group showed significantly reduced HIF-1α expression compared to both the Air and O_3_ groups, indicating that hydrogen gas effectively attenuated the cellular hypoxic response (Figure 3B,C).

These results suggest that hydrogen gas not only reduces tissue hypoxia, as indicated by LOX-1 and HIF-1α expression, but also improves vascular integrity in the medaka brain.

### 3.3. Hydrogen Gas Suppresses Hypoxia-Induced Apoptotic Cell Death and Oxidative DNA Damage in the Medaka Brain Tissues

**Figure 4:**
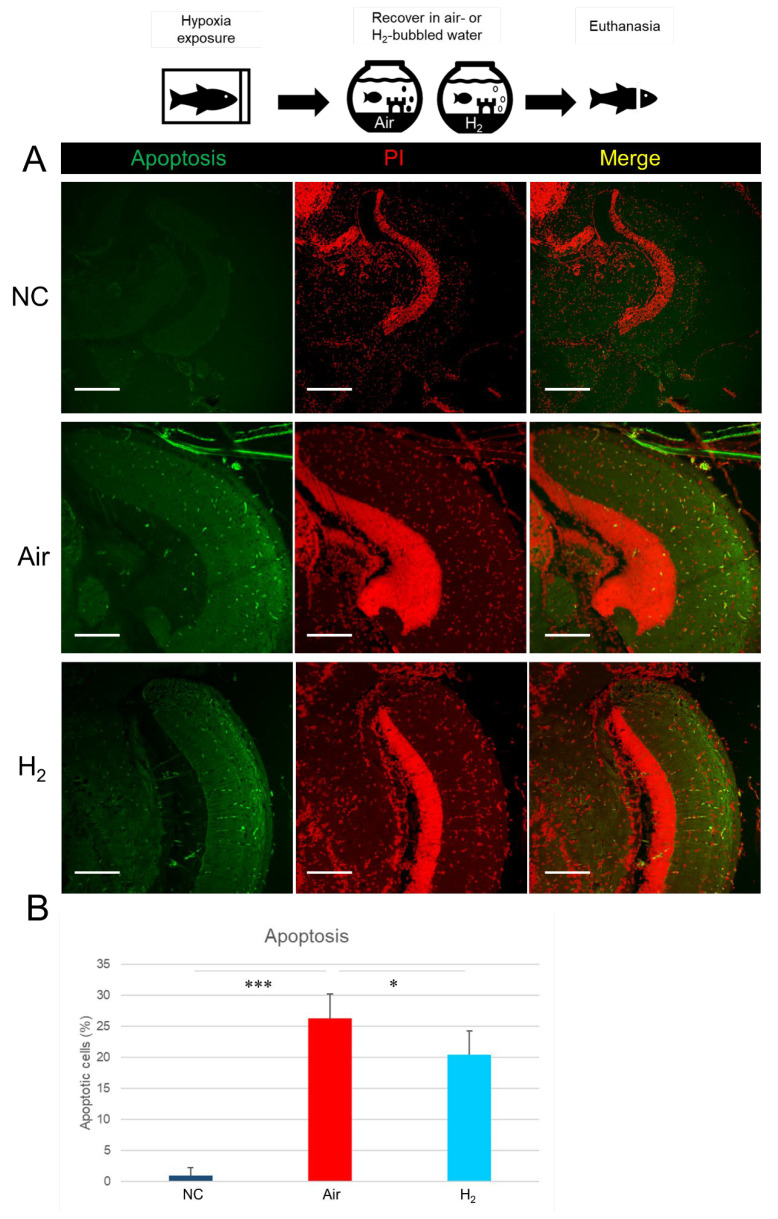
Effect of hydrogen gas on hypoxia-induced apoptosis in medaka brain tissues. Matsushiro medaka (*n* = 20/group) was exposed to a hypoxic environment for 9 min, then recovered for 1 h in either air-bubbled water or hydrogen gas-bubbled water, as illustrated in the top panel. Brain sections were stained using a TUNEL assay kit and examined with a laser scanning microscope. (**A**), Representative images of TUNEL-stained optic tectum of the brain sections. Two additional examples for each group are provided in Supplemental Figure S5. Scale bar = 100 µm. Green, apoptotic cells, Red, nuclei stain by PI (propidium iodide). (**B**), Quantification of apoptotic cells in the optic tectum from 10 sections per group using ImageJ software. * *p* < 0.05, *** *p* < 0.001. Experiments were independently repeated three times.

We next investigated whether H_2_ could inhibit hypoxia-induced apoptosis and oxidative DNA damage in the brain tissues of medaka. TUNEL assay (Figure 4A,B) revealed that apoptotic cell death was rarely observed in the optic tectum of the NC group, while it was significantly increased in the Air group. In contrast, H_2_ treatment significantly reduced the number of apoptotic cells compared to the Air group.

**Figure 5:**
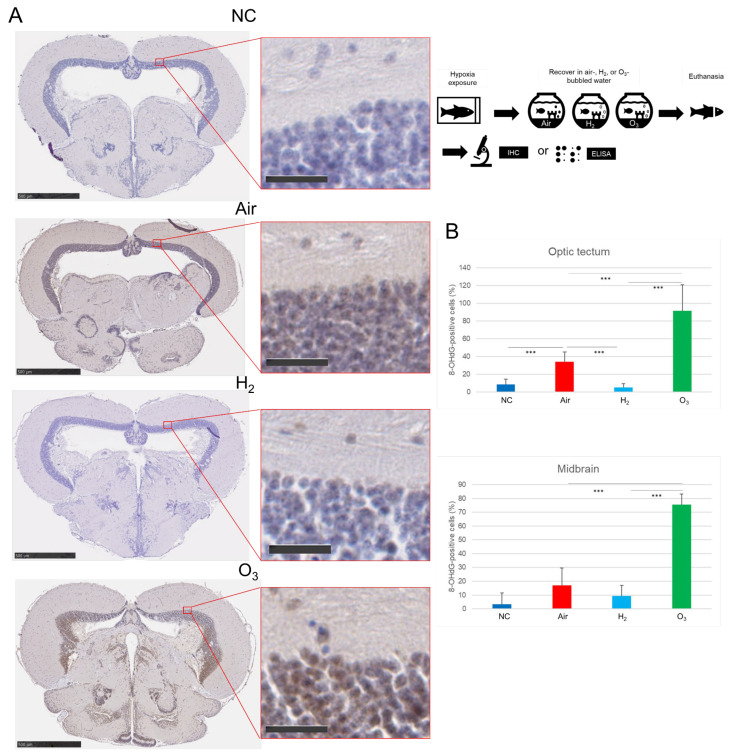
Effect of hydrogen gas on hypoxia-induced oxidative DNA damage in medaka brain tissues. As illustrated in the upper right panel, red medaka (*n* = 20) was exposed to hypoxia and recovered under the same conditions described in Figure 3. Brain sections were subjected to IHC staining with an anti-8-OHdG antibody and scanned using a NanoZoomer S20. (**A**), Representative images of 8-OHdG-stained brain sections. Two additional examples for each group are provided in Supplemental Figure S6. Brown indicates positive 8-OHdG staining, while blue indicates nuclear counterstaining. Left: whole brain slice (scale bar = 500 µm). Right: enlarged view of the optic tectum (scale bar = 25 µm). (**B**), Quantification of 8-OHdG-positive cells in the optic tectum from 10 sections per group using ImageJ software. *** *p* < 0.001. Experiments were independently repeated five times.

To assess oxidative DNA damage, we evaluated 8-OHdG expression in the brain using IHC. IHC staining showed minimal 8-OHdG expression in the midbrains of the NC group (Figure 5A,B). However, both the Air and O_3_ groups exhibited widespread 8-OHdG-positive cells in the optic tectum and midbrain core, with the O_3_ group showing the most intense staining. In contrast, the H_2_ group showed significantly fewer 8-OHdG-positive cells than both the Air and O_3_ groups.

These findings suggest that hydrogen gas protects medaka brain tissue from hypoxia-induced apoptosis and oxidative DNA damage under hypoxic conditions.

### 3.4. Hydrogen Gas Inhibits Hypoxia-Induced Inflammatory Changes in Medaka Brain Tissues

**Figure 6:**
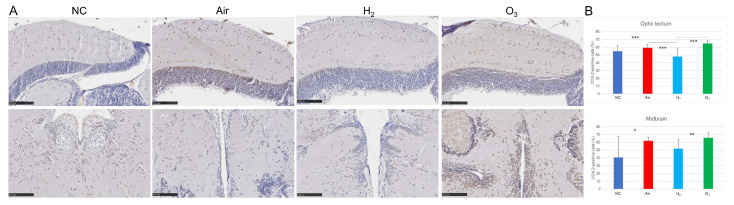
Effect of hydrogen gas on hypoxia-induced COX-2 expression in medaka brain. Red medaka (*n* = 20/group) was exposed to hypoxia and recovered under the same conditions described in Figure 3. Brain sections were subjected to IHC staining with an anti-COX-2 antibody and scanned using a NanoZoomer S20. (**A**), Representative images of COX-2-stained brain sections. Two additional examples for each group are provided in Supplemental Figure S7. Brown indicates positive COX-2 staining, while blue indicates nuclear counterstaining. Scale bar = 100 µm. (**B**), Quantification of COX-2-positive cells in the optic tectum (upper panel) and the midbrain (lower panel) from 10 sections per group using ImageJ software. * *p* < 0.05, **, *p* < 0.01, *** *p* < 0.001. Experiments were independently repeated five times.

We further examined the effects of H_2_ on hypoxia-induced inflammatory responses in medaka brain tissues using IHC and ELISA. Immunostaining for cyclooxygenase-2 (COX-2), an inducible pro-inflammatory enzyme, revealed a marked increase in COX-2 expression in the optic tectum of both the Air and O_3_ groups (Figure 6A,B). In the midbrain core, COX-2 expression was significantly elevated in the O_3_ group compared to the negative control, whereas the Air group showed a moderate, non-significant increase. In contrast, COX-2 expression in the H_2_ group was significantly lower than in both the Air and O_3_ groups, particularly in the optic tectum region.

**Figure 7:**
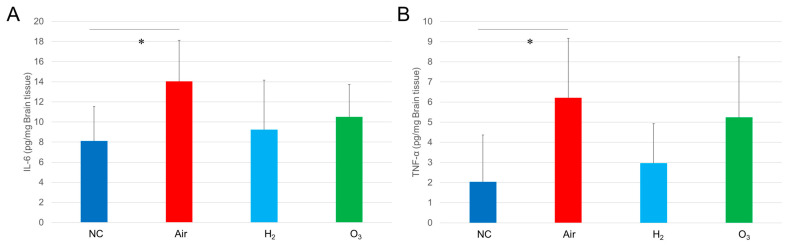
Effect of hydrogen gas on hypoxia-induced inflammatory cytokine secretion in medaka heart tissues. Red medaka (*n* = 20/group) was exposed to hypoxia and recovered under the same conditions described in Figure 3. The fish brain tissues were collected and homogenized using an ultrasonic homogenizer and processed with a RIPA protein extraction kit, followed by analysis using IL-6 and TNF-α ELISA kits. (**A**), IL-6 ELISA results for brain tissues. (**B**), TNF-α ELISA results for brain tissues. * *p* < 0.05. Experiments were independently repeated five times.

ELISA results revealed that in brain tissues, IL-6 and TNF-α levels were significantly increased only in the Air group. Although H_2_ treatment tended to lower both IL-6 and TNF-α levels in the brain, the differences among the H_2_, Air, and O_3_ groups were not statistically significant (Figure 7A,B).

These findings suggest that hydrogen gas can suppress hypoxia-induced inflammatory changes, partially in the brain, under acute hypoxic conditions in medaka.

### 3.5. Hydrogen Gas Restores Antioxidant Capacity and Normalizes Respiratory and Locomotor Activity Following Hypoxia

Brain injuries are known to disrupt enzyme activity, compromise antioxidant defenses, and cause respiratory and behavioral changes [32,33]. To examine whether acute hypoxia induces similar effects in medaka, we measured acetylcholinesterase (AChE) activity, total antioxidant capacity, respiratory rate, and locomotor behavior following hypoxic exposure and recovery under different gas conditions.

**Figure 8:**
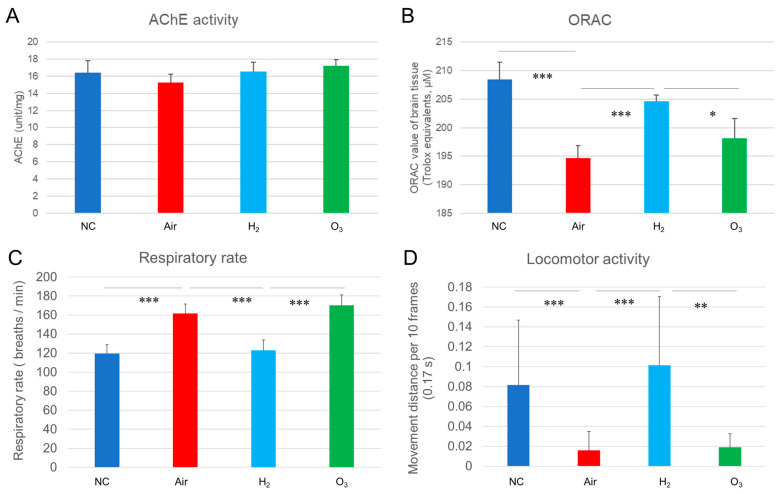
Effect of hydrogen gas on respiratory rate, locomotor activity, AChE activity, and antioxidant capacity in medaka brain tissues following hypoxic exposure. Black medaka (*n* = 20 per group) was exposed to acute hypoxia and allowed to recover under the same conditions described in Figure 3. After recovery, the fish were euthanized, and brain tissues were collected and homogenized in ice-cold 0.05 M phosphate buffer. Supernatants were used to measure AChE activity and antioxidant capacity using the ORAC assay. Recovery behavior was recorded, and a representative excerpt is shown in Supplemental Video S2. For analysis, longer video recordings were processed using Tracker software to assess opercular (breathing) rate and locomotor activity. (**A**), AChE activity in brain tissues. (**B**), ORAC values indicating antioxidant capacity. (**C**), Respiratory rate following hypoxia exposure. (**D**), Locomotor activity quantified as movement distance per 0.17 s (10 frames). * *p* < 0.05, ** *p* < 0.01, *** *p* < 0.001. All experiments were independently repeated three times.

As shown in Figure 8A, AChE activity did not significantly differ among the four groups, suggesting that a single episode of acute hypoxia did not induce detectable changes in cholinergic enzyme function within the one-hour recovery period. It is possible that longer or repeated exposures are needed to affect AChE activity.

In contrast, the ORAC assay revealed a significant reduction in antioxidant capacity in the Air and O_3_ groups compared to the NC group (Figure 8B), indicating that hypoxia-induced oxidative stress exceeded the brain’s intrinsic antioxidant defenses. Hydrogen gas treatment significantly restored antioxidant capacity to levels comparable with the NC group, highlighting its protective effect against oxidative damage.

To evaluate functional recovery, we measured opercular (gill) movement frequency and locomotor activity during the recovery period using Tracker software (representative behavior shown in Supplemental Video S2). As shown in Figure 8C, the respiratory rate increased to 162 and 170 breaths per minute in the Air and O_3_ groups, respectively, compared to 120 breaths per minute in the NC group. In contrast, fish in the H_2_ group showed a rate of 123 breaths per minute, closely resembling the NC group and significantly lower than both the Air and O_3_ groups.

Locomotor analysis (Figure 8D) demonstrated a sharp reduction in movement distance in the Air (0.016 cm/10 frames) and O_3_ (0.019 cm/10 frames) groups, compared to 0.082 cm/10 frames in the NC group. Remarkably, fish in the H_2_ group exhibited a movement distance of 0.101 cm/10 frames, significantly higher than both the Air and O_3_ groups and comparable to the NC group.

These results suggest that hydrogen gas not only restores antioxidant capacity but also enhances respiratory function and behavioral performance following acute hypoxic injury, indicating improved overall physiological resilience in medaka.

## 4. Discussion

This study demonstrates that acute hypoxic exposure causes significant brain injury in medaka, as evidenced by cerebral hemorrhage, vascular congestion, neuronal apoptosis, oxidative DNA damage, reduced antioxidant capacity, elevated inflammatory responses, and alterations in respiratory rate and locomotor activity. Importantly, recovery in hydrogen gas-bubbled water markedly attenuated these hypoxia-induced effects, highlighting hydrogen’s potential as a neuroprotective agent.

We also demonstrated that opercular movement, a proxy for respiratory rate in fish, is a sensitive indicator of hypoxic stress. The biphasic response—initial hyperventilation followed by a sharp decline—paralleled the progression from mild to severe hypoxia. These physiological changes were accompanied by cerebral hemorrhage, vascular congestion and apoptotic cell death, particularly in the midbrain, which mirror the vascular disruptions and neuronal damage reported in mammalian models of hypoxic–ischemic encephalopathy [34].

Hydrogen gas treatment significantly mitigated vascular pathology and neuronal apoptosis. H&E staining and TUNEL assays showed reduced vascular congestion and apoptotic cell numbers in the midbrain. These histological improvements were mirrored by enhanced locomotor activity and stabilized respiratory rates in hydrogen-treated fish, suggesting better systemic recovery.

At the molecular level, strong LOX-1 and HIF-1α signals confirmed persistent hypoxic injury in the brain, even after one hour of recovery in air-bubbled water. Histological evidence of vascular congestion suggests that secondary hypoxia, resulting from impaired blood flow, may have contributed to the sustained brain injury. In contrast, hydrogen gas markedly reduced vascular congestion and suppressed LOX-1 and HIF-1α signals, indicating improved tissue oxygenation or mitigation of hypoxia-induced cellular stress. These results are consistent with previous findings showing that hydrogen modulates HIF-1α signaling and alleviates hypoxia-related tissue damage [35].

Oxidative stress, a hallmark of hypoxia-induced injury, was evident through increased 8-OHdG levels and reduced ORAC values in the brain. Hydrogen treatment effectively reversed these changes, reflecting its ability to neutralize hydroxyl radicals and peroxynitrite [36]. Additionally, hydrogen reduced COX-2 expression and suppressed inflammatory cytokines (IL-6 and TNF-α), reinforcing its anti-inflammatory potential as reported in clinical and preclinical studies [37,38].

Notably, ozone gas exacerbated hypoxia-induced damage across multiple parameters, including vascular congestion, oxidative DNA damage, inflammation, elevated respiratory rate, and reduced locomotor activity. Owing to its strong oxidative potential, ozone likely intensifies tissue injury by reacting with cellular macromolecules. In contrast, H_2_, due to its extremely small molecular size and nonpolar nature, readily diffuses across biomembranes—including the blood–brain barrier, cytosol, mitochondria, and nuclei—enabling deep tissue penetration and effective scavenging of reactive oxygen species.

The neuroprotective effects of hydrogen gas against acute hypoxia-induced brain injury likely arise from its pleiotropic actions. First, hydrogen selectively reduces the most cytotoxic reactive oxygen species (•OH and ONOO^−^) without interfering with physiological ROS required for cellular signaling, thereby restoring redox balance [39]. Second, hydrogen has been reported to regulate mitochondrial function, maintaining ATP production and limiting mitochondrial permeability transition, which are crucial during hypoxic stress [40]. Third, suppression of HIF-1α and hypoxia-induced vascular congestion observed in this study suggests that hydrogen may stabilize vascular function and prevent hypoxia-induced endothelial dysfunction. Fourth, hydrogen’s anti-inflammatory effects, achieved through downregulation of NF-κB and proinflammatory cytokines [41], may further limit secondary tissue injury. Collectively, these multifaceted mechanisms could explain the broad protective outcomes observed in medaka.

Despite these promising findings, several limitations should be acknowledged. First, our study was performed in medaka, a small teleost fish, which—while valuable for modeling hypoxia and neurovascular responses—may not fully recapitulate mammalian brain complexity. Second, the exact kinetics of hydrogen uptake and distribution in medaka tissues were not quantified; future studies should measure hydrogen concentrations in brain tissue to confirm bioavailability. Third, although we demonstrated reductions in oxidative stress and inflammation, the precise molecular signaling pathways modulated by hydrogen (e.g., mitochondrial signaling, MAPK, Nrf2 activation) were not directly examined. Fourth, we tested only acute hypoxic exposure, and the long-term effects of repeated hypoxia and hydrogen treatment remain unexplored. Finally, translation of findings from aquatic models to clinical practice requires cautious interpretation, as differences in physiology and exposure methods may influence outcomes.

Together, these findings highlight hydrogen gas as a promising therapeutic agent against acute hypoxia-induced brain injury. Its multifaceted benefits—including preservation of vascular integrity, inhibition of apoptosis, suppression of oxidative and inflammatory stress, and enhancement of functional recovery—support its further investigation in both experimental and clinical settings. Addressing the above limitations through mechanistic studies and mammalian validation will be essential to advancing hydrogen-based therapies for hypoxic brain injury.

## 5. Conclusions

This study demonstrates that hydrogen gas confers significant protective effects against acute hypoxia-induced brain injuries in medaka. Hydrogen treatment reduced vascular congestion, neuronal apoptosis, oxidative DNA damage, and inflammation, while restoring antioxidant capacity and promoting functional recovery. In contrast, ozone exposure exacerbated hypoxic injuries, underscoring the importance of carefully selecting therapeutic gases in hypoxic conditions. These findings support the potential of hydrogen gas as a safe and effective intervention for preventing or mitigating hypoxia-related tissue damages and warrant further investigation in higher vertebrate models and clinical studies.

## Data Availability

The data that support the findings of this study are available within the article. Other data related to this study are available upon request from the corresponding author.

## Supplementary Materials

The following supporting information can be downloaded at: https://www.mdpi.com/article/10.3390/antiox14091130/s1, Supplemental Video S1. Hypoxia Exposure of Medaka, Supplemental Video S2. Recovery behavior of medaka under different conditions, Supplemental Figure S1. TTC staining depends on intact brain tissue in medaka, Supplemental Figure S2. Prolonged hypoxia exposure induces brain damage in medaka. Supplemental Figures S3–S7. Two additional representative images for each treatment group corresponding to Figures S2–S6.
