# Supplementary figures and images for: Hydrogen Gas Mitigates Acute Hypoxia-Induced Oxidative and Inflammatory Brain Injuries in Medaka (Oryzias latipes)
Source: Antioxidants (Basel). 2025 Sep 18;14(9):1130. doi: 10.3390/antiox14091130 (PMC12466362; doi:10.3390/antiox14091130)

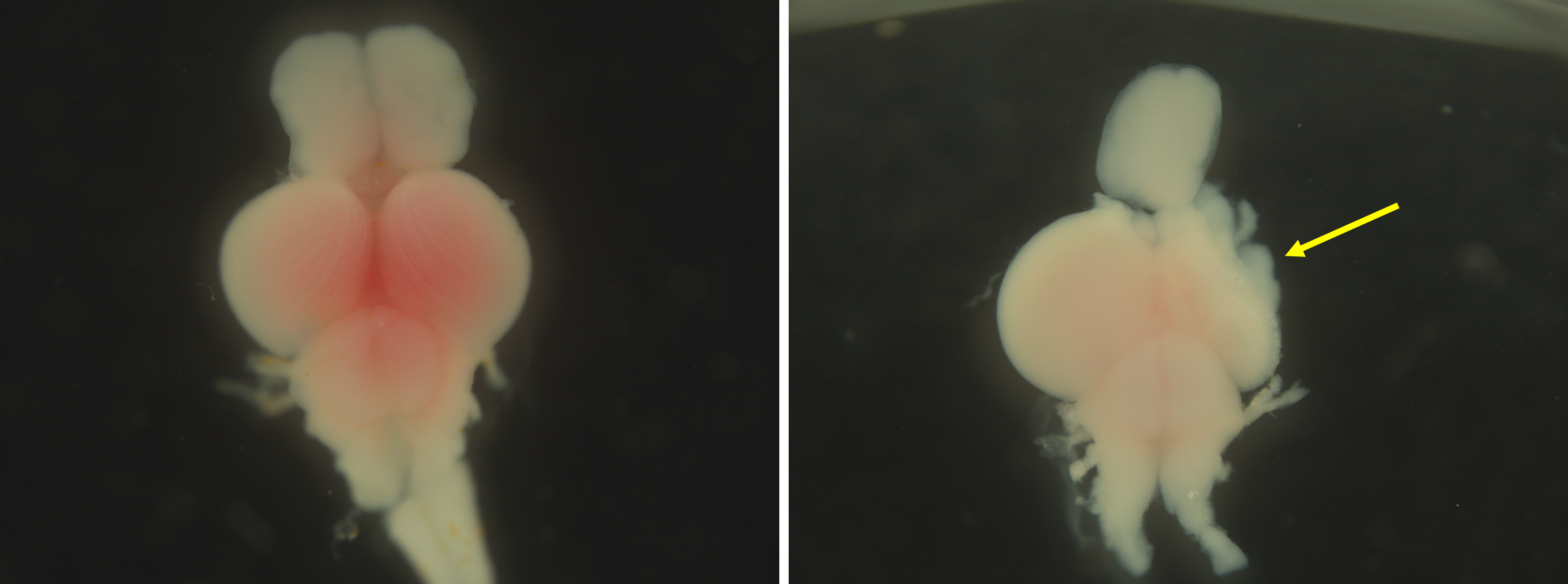

Supplement: Supplementary file 1 [file antioxidants-14-01130-s001.zip › antioxidants-3834752-supplementary/Sup Fig 1.tiff]

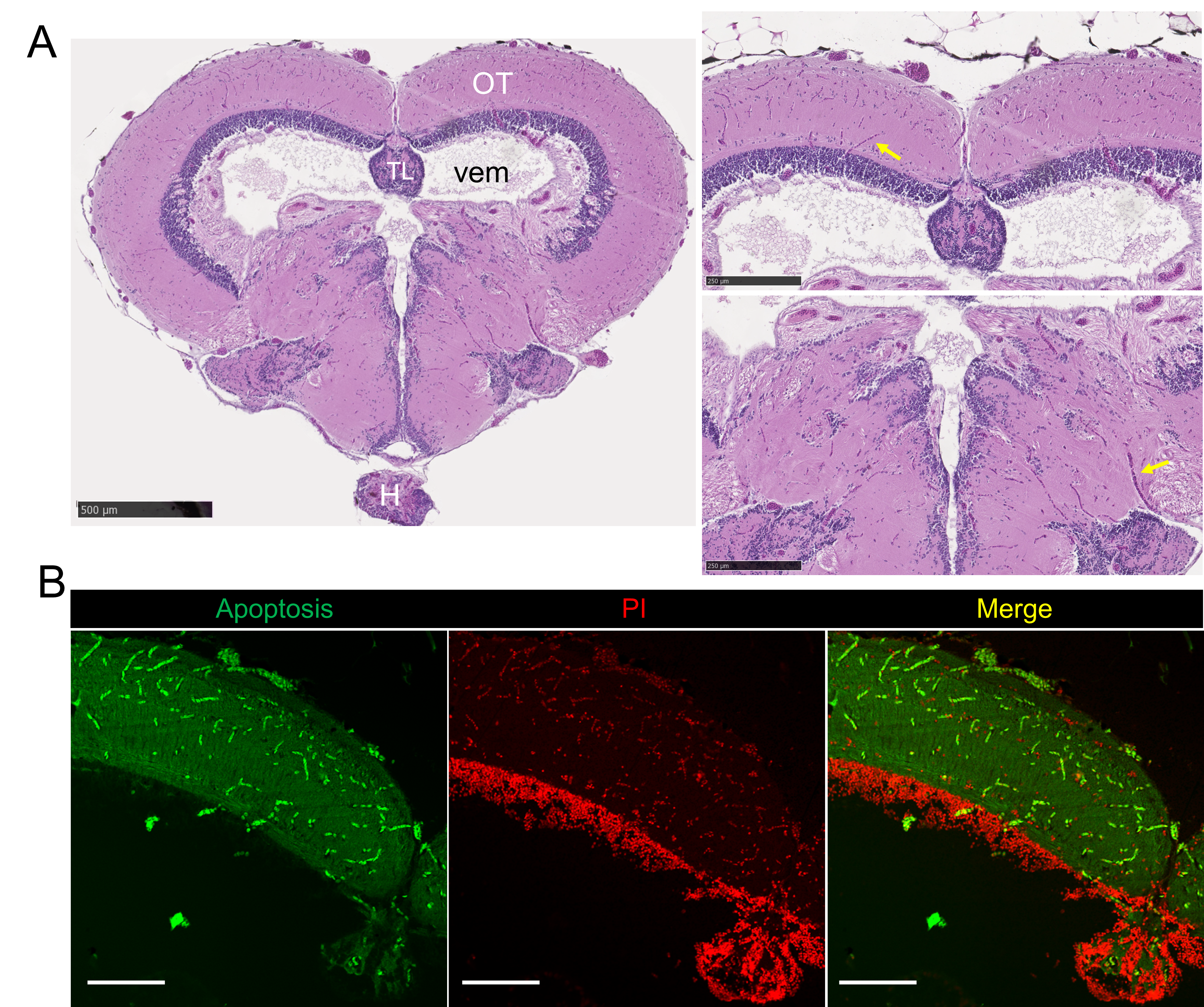

Supplement: Supplementary file 1 [file antioxidants-14-01130-s001.zip › antioxidants-3834752-supplementary/Sup Fig 2.tiff]

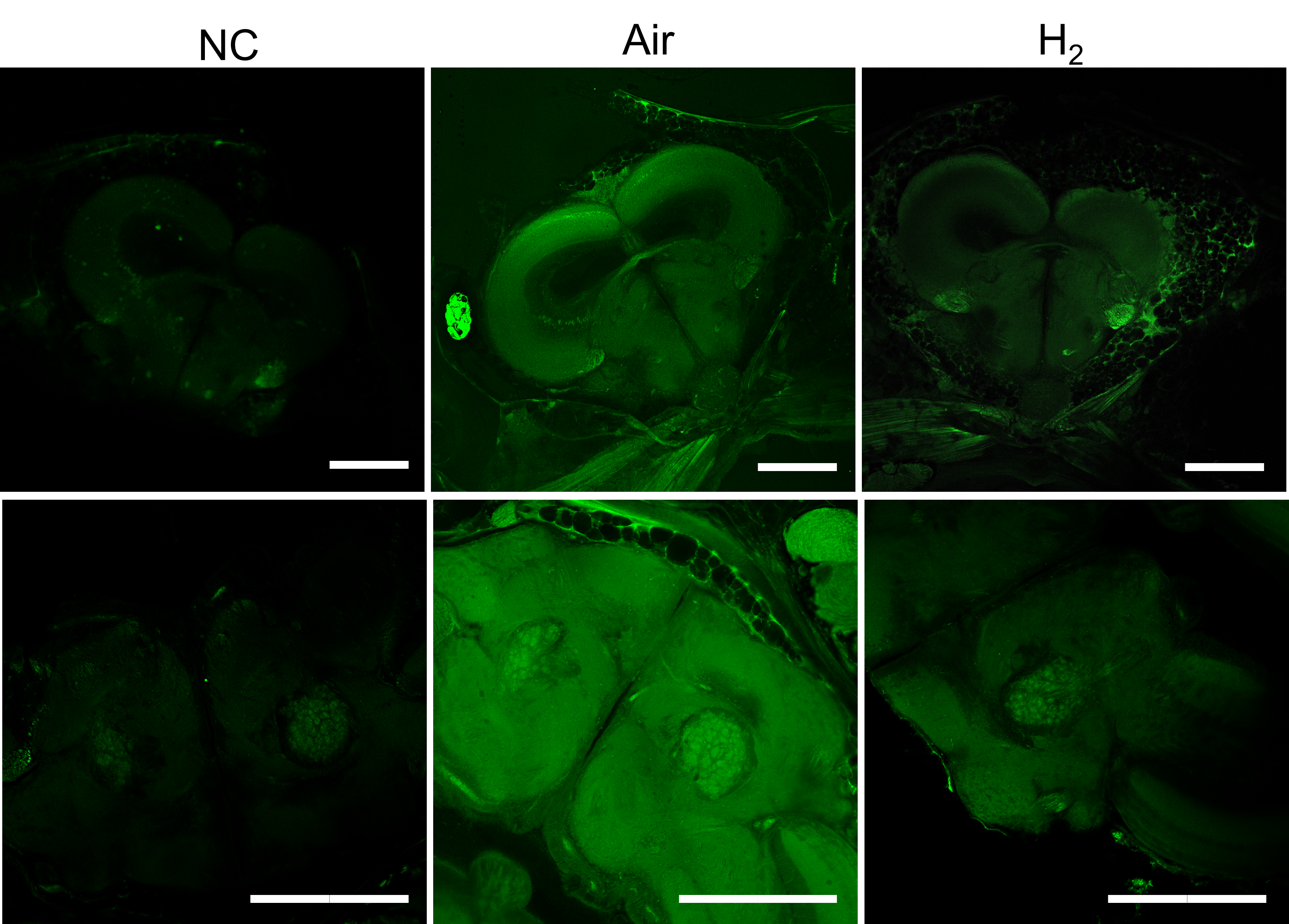

Supplement: Supplementary file 1 [file antioxidants-14-01130-s001.zip › antioxidants-3834752-supplementary/Sup Fig 3.tiff]

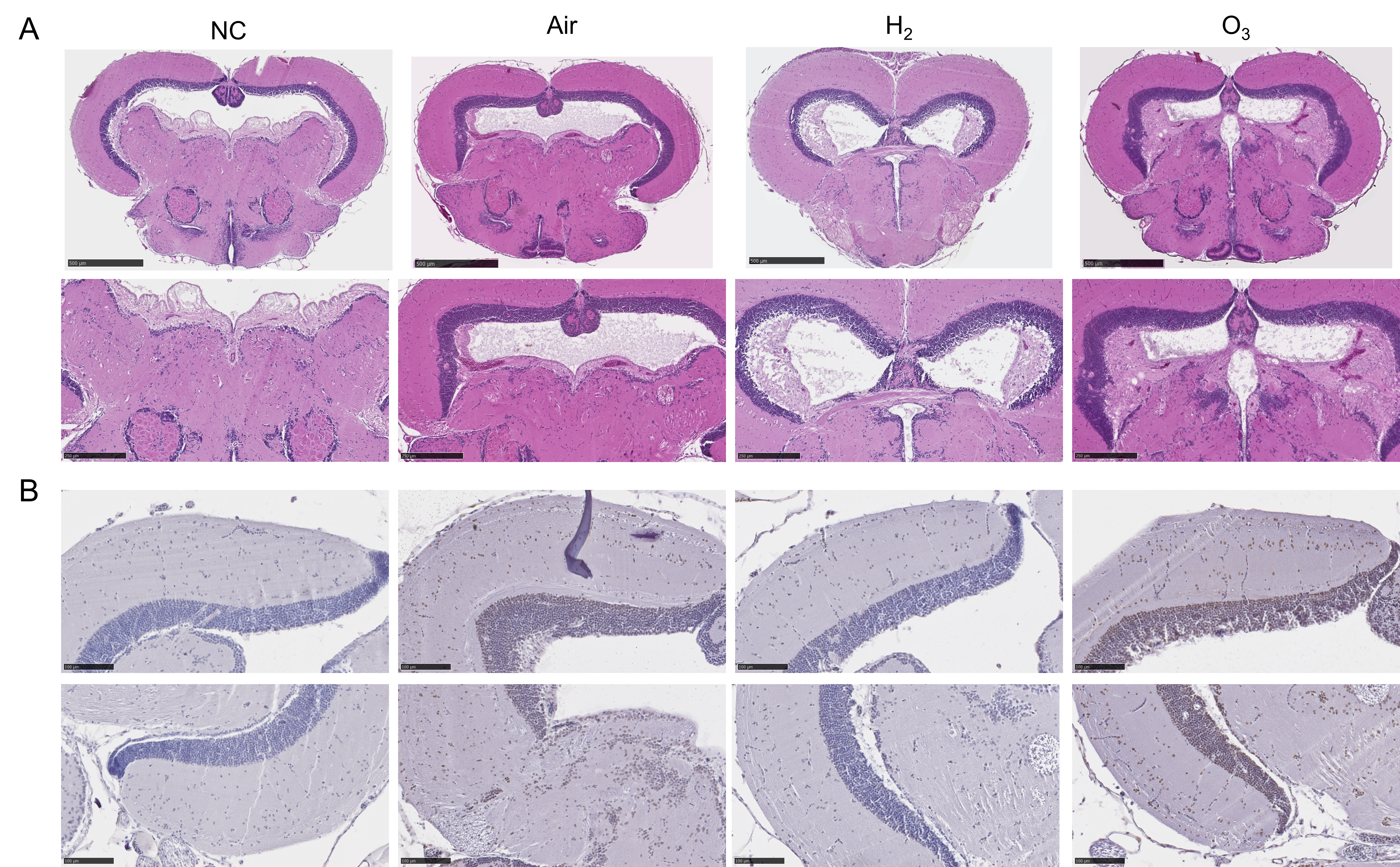

Supplement: Supplementary file 1 [file antioxidants-14-01130-s001.zip › antioxidants-3834752-supplementary/Sup Fig 4.tiff]

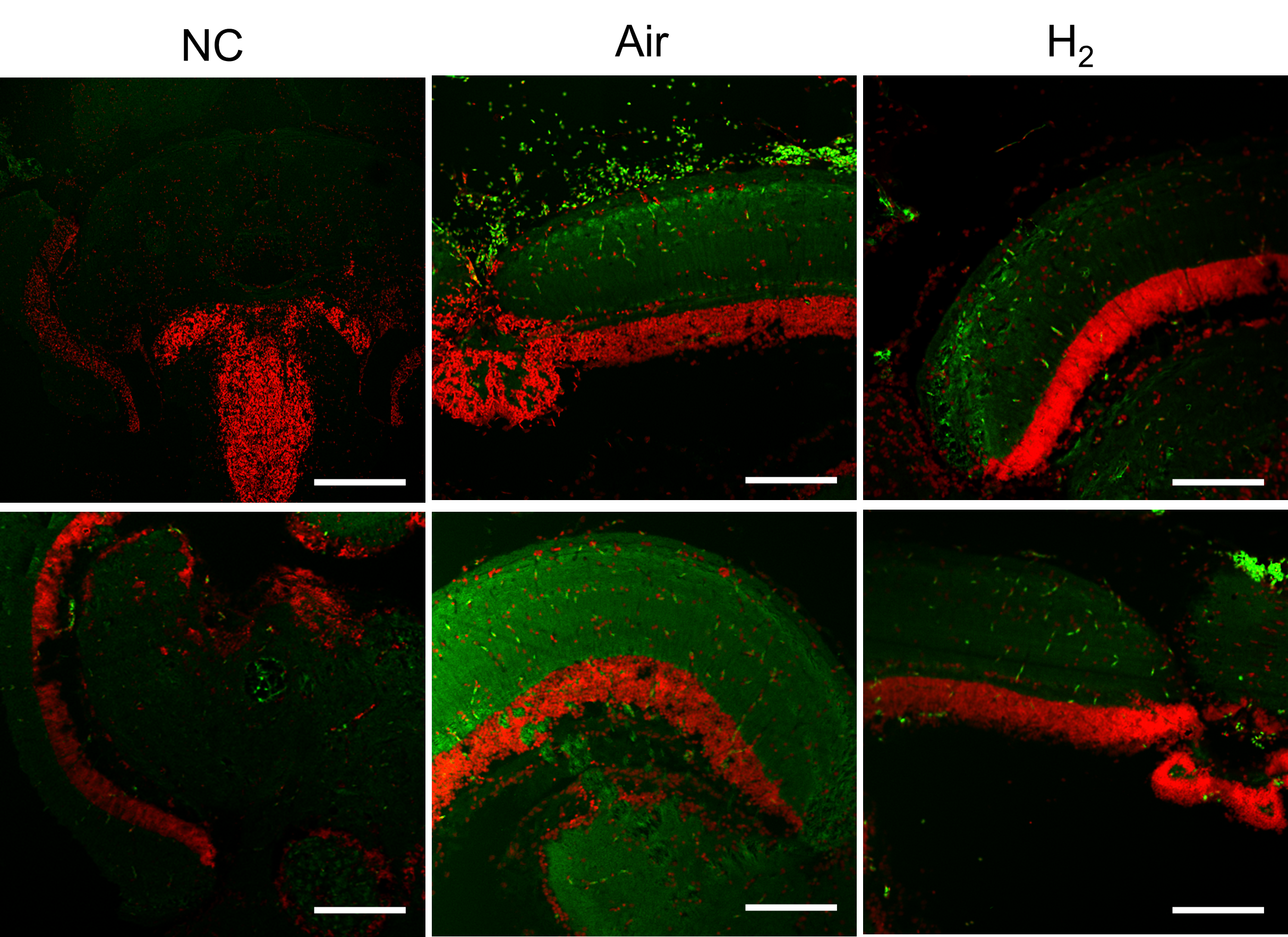

Supplement: Supplementary file 1 [file antioxidants-14-01130-s001.zip › antioxidants-3834752-supplementary/Sup Fig 5.tiff]

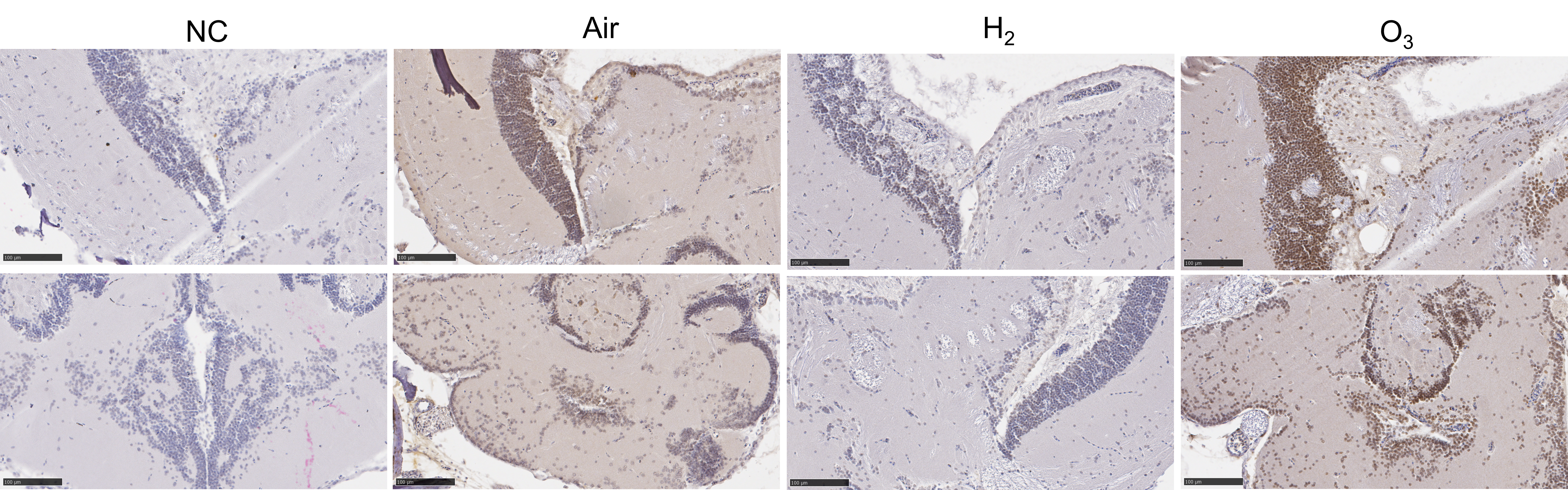

Supplement: Supplementary file 1 [file antioxidants-14-01130-s001.zip › antioxidants-3834752-supplementary/Sup Fig 6.tiff]

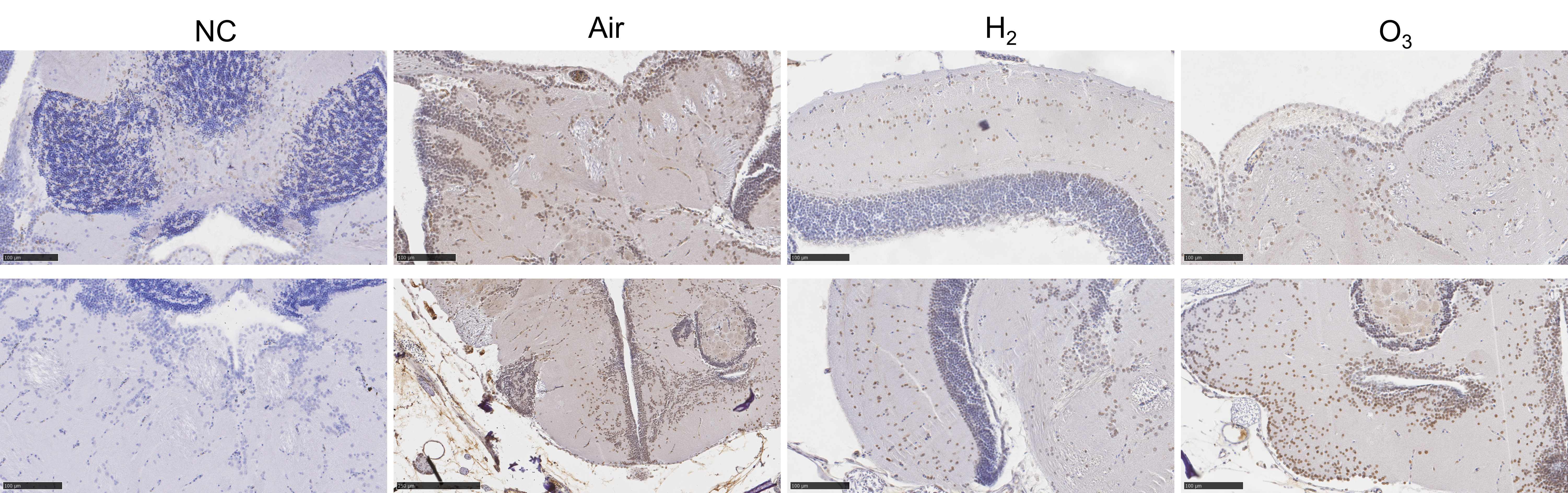

Supplement: Supplementary file 1 [file antioxidants-14-01130-s001.zip › antioxidants-3834752-supplementary/Sup Fig 7.tiff]
