# Supplementary material for: Hydrogen Gas Mitigates Acute Hypoxia-Induced Oxidative and Inflammatory Brain Injuries in Medaka (Oryzias latipes)
Source: Antioxidants (Basel). 2025 Sep 18;14(9):1130. doi: 10.3390/antiox14091130 (PMC12466362; doi:10.3390/antiox14091130)
Supplement: Supplementary file 1 [file antioxidants-14-01130-s001.zip › antioxidants-3834752-supplementary/antioxidants-3834752-Supplemental data.docx]

Supplemental data

**Hydrogen Gas Mitigates Acute Hypoxia-Induced Oxidative and Inflammatory Brain and Heart Injuries in Medaka (*Oryzias latipes*)**

Eriko Sato^1, 2^, Naohiro Shimamura^2^, Chikako Saiki^1^, Katsuhisa Sunada^2^, Nobuhiko Miwa^3,4^, Li Xiao^1*^

1 Department of Physiology, School of Life Dentistry at Tokyo, The Nippon Dental University, 1-9-20 Fujimi, Chiyoda-ku, Tokyo 102-8159, Japan

2 Department of Dental Anesthesiology, School of Life Dentistry at Tokyo, The Nippon Dental University

3 Incorporated Association Hydrogen Medical Institute, Minatojima Minamicho 1-6-4, ChuOh-Ku, Kobe 650-0047, Japan

4 Prefectural University of Hiroshima, Faculty of Life Sciences, Hiroshima 727-0023, Japan

*Correspondence to:

Li Xiao, MD, PhD, [xiaoli@tky.ndu.ac.jp](mailto:xiaoli@tky.ndu.ac.jp)

**Materials and Methods**

*TTC Staining*

Brain viability was assessed using 2,3,5-triphenyltetrazolium chloride (TTC, Sigma-Aldrich). Freshly excised brains were immersed in 0.4% TTC in PBS (-) for 30 minutes at room temperature, then fixed in 10% neutral buffered formalin. Images were captured using a stereo microscope (AxioZoom.V16, Carl Zeiss).

**Supplemental Video 1. Hypoxia exposure of medaka and measurement of dissolved oxygen.**

Adult medaka were subjected to hypoxia by placement in a Ziplock bag containing minimal air and water. Opercular movements (respiratory rate) were recorded using a TOMLOV digital microscope (first 20 s segment). Dissolved oxygen was measured by inserting a probe into the same bag, which was additionally enclosed within two larger Ziplock bags to prevent air leakage. Oxygen levels were monitored continuously for 18 min, and a 10 s segment from 8–9 min of the recording is shown.

**Supplemental Figure 1. TTC staining depends on intact brain tissue in medaka.**
Black medaka (n = 6) without any hypoxia treatment (negative control) were euthanized, and their brains were immediately dissected and stained with freshly prepared TTC solution. Intact brains showed uniform red staining (left), whereas mechanically damaged brains exhibited pale or unstained regions. The yellow arrow indicates an area of dissection-induced injury.
Experiments were independently repeated three times.

**Supplemental Figure 2. Prolonged hypoxia exposure induces brain damage in medaka.**
Matsushiro medaka (n=3) were exposed to a hypoxic environment for more than 30 minutes. After exposure, the fish were euthanized, and brain tissues were fixed in Davidson solution, dehydrated, and embedded in paraffin. Paraffin sections were stained with H&E or subjected to TUNEL assay, and examined using a laser scanning microscope.
**A**, Representative H&E-stained sections of the midbrain. Yellow arrows indicate congested blood vessels. Scale bars: 500 µm (left), 100 µm (right).
**B**, Representative TUNEL-stained images of the optic tectum. Scale bar = 100 µm.
Experiments were independently repeated three times.

**Supplemental Figure 3.** Additional representative images of LOX-1 fluorescence in medaka brain slices corresponding to Figure 2. Scale bar = 200 µm.

**Supplemental Figure 4.** A, Additional representative images of H&E staining in medaka brain slices corresponding to Figure 3. Scale bar = 500 µm (upper panel) and 100 µm (lower panel). B, Additional representative images of HIF-1α immunostaining in medaka brain slices corresponding to Figure 3. Scale bar = 100 µm.

**Supplemental Figure 5.** Additional representative images of TUNEL staining in medaka brain slices corresponding to Figure 4. Scale bar = 100 µm.

**Supplemental Figure 6.** Additional representative images of IHC staining with an anti-8-OHdG antibody in medaka brain slices corresponding to Figure 5. Scale bar = 100 µm.

**Supplemental Figure 7.** Additional representative images COX-2 immunostaining in medaka brain slices corresponding to Figure 6. Scale bar = 100 µm.

Supplemental Video 2. Recovery behavior of medaka under different conditions.

Black medaka were exposed to acute hypoxia and then allowed to recover under the same conditions described in Fig. 3. This edited video serves as a representative example of the recovery process. The video begins with a 10-second segment showing fish under hypoxic conditions, followed by 10 seconds of the NC group maintained in normal breeding water. Subsequent segments show typical behavior of fish recovering for 20 minutes in Air, H₂, and O₃ water. The longer original recordings used for movement analysis were processed separately using Tracker software.
